# Analysis of Volatile Components From Different Commercially Available Fenugreek Tinctures Based on GC‐IMS and SPME‐GC–MS and Their Correlation With Sensory Aroma

**DOI:** 10.1002/fsn3.4763

**Published:** 2025-04-04

**Authors:** Hua Zhang, Haifeng Shen, Yuanqing Ye, Fan Cao, Jiale Ren, Huaiyuan Zhu, Bo Chi, Huiyun Liao, Feng Li

**Affiliations:** ^1^ China Tobacco Jiangsu Industrial Co., Ltd. Nanjing China; ^2^ State Key Laboratory of Materials‐Oriented Chemical Engineering, College of Biotechnology and Pharmaceutical Engineering, College of Food Science and Light Industry Nanjing Tech University Nanjing China; ^3^ China Tobacco Guangdong Industrial Co., Ltd. Guangzhou China

**Keywords:** fenugreek tincture, GC‐IMS, key flavor components, OAV, SPME‐GC–MS

## Abstract

This study explored the key flavor components of fenugreek tinctures from various manufacturers and how these components affect the aromas. Headspace solid‐phase microextraction‐gas chromatography–mass spectrometry (SPME‐GC–MS) and headspace gas chromatography‐ion mobility spectrometry (GC‐IMS) were used to identify the volatile components. The key flavor components were determined by descriptive sensory analysis, multivariate statistical analysis, and odor activity value (OAV). Further analysis was conducted on the correlation for these components with sensory aroma characteristics. The results indicated that the principal aromas of fenugreek tincture samples were burnt and herbal aroma with additional sweet, hay, and balsamic aroma. A total of 148 compounds were identified by GC‐IMS and SPME‐GC–MS including nine key flavor components determined. It was found that benzaldehyde, n‐butyraldehyde, propyl butyrate, 3‐ethyl‐2,5‐dimethylpyrazine, and 2,5‐dimethylpyrazine were positively correlated with burnt, baking, and herb aroma while N‐butyraldehyde was significantly positively correlated with freshness aroma and significantly negatively correlated with spicy aroma. Benzaldehyde was determined to be significantly positively correlated with sweet aroma while 3‐ethyl‐2,5‐dimethylpyrazine and 2,5‐dimethylpyrazine were significantly positively correlated with herbal aroma. The findings of this study can provide indications for quality identification, origin traceability, and extraction process optimization of fenugreek tincture products.

## Graphical abstract

This study examines the variations in the key volatile flavor compounds of several fenugreek tincture samples from various sources and their relationship with flavor characterization using GC‐IMS, SPME‐GC–MS, OAV, and DSA. This study elaborates on the intrinsic association between key flavor components and sensory flavor, as well as the contributions of key components to the overall flavor of fenugreek tincture. This work can provide a technical views for future research on the flavor and sensory attributes of fenugreek tincture.

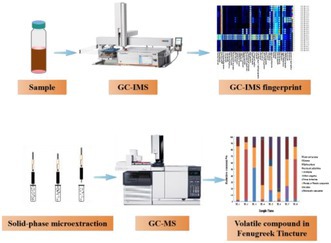

## 1 Introduction

Fenugreek tincture is a liquid phase extract made by ethanol extraction from the mature and dried seeds of the leguminous Fenugreek plant, which belongs to the Papilionaceous subfamily (Ebubekir, Engin, and Faruk 2004; Zhou, Chan, and Zhou 2012; Aasim et al. 2018). It smells like caramel and maple with a faint foot odor and a nutty scent as well. Fenugreek tincture plays a significant role in natural fragrance materials and thus it is frequently used as a spice and food additive (Huang 1999, 2005; Su et al. 2012; Olaiya and Soetan 2014). Significant differences were found among different commercial fenugreek tincture products due to of the variation in raw materials and extraction procedure used in different manufacturers. Thus, these variations have an significant impact on the quality control, identification of active ingredients, and application in food science (Zandi et al. 2017; Qamar et al. 2020). Recent studies of fenugreek tincture are very systematic, but mainly emphasizing on the nonvolatile components (An et al. 2010; Reddy, Gowda, and Srinivasan 2019; Wani and Kumar 2018; Vi et al. 2024). Several groups have investigated the volatile ingredients from fenugreek tincture samples, but there are no further explorations on the flavor quality and its process control techniques (Ebubekir, Engin, and Faruk 2004; Huang 2005; Dong et al. 2004). As the primary contributor to the flavor features of fenugreek tincture are mostly volatile components it is crucial to investigate the volatile flavor ingredients in fenugreek tincture and how they contribute to distinctive aroma. This may help to set up a systematic evaluation process for the determination of fenugreek tincture products quality, extraction method optimization, and application efficacy improvement (Su, Mao, Li, et al. 2012; Su, Mao, Qu, et al. 2012; Dong et al. 2004).

Gas chromatography ion mobility spectroscopy (GC‐IMS) combines the high separation ability of GC with the rapid response of IMS (Wang, Chen, and Sun 2020). Its advantages include a low detection limit, fast analysis, no sample preparation requirements, and ease of use. Therefore, GC‐IMS have been widely used in flavor components determination for fruits, meat, fish, alcoholic drinks, biological fermentation, and edible oil (Xu et al. 2024; Zhao et al. 2021; Wu et al. 2022, 2023; Zeng et al. 2022; Chen et al. 2024; Xiao et al. 2022; Hao et al. 2024; Liu et al. 2022; Zhu et al. 2018; Chang et al. 2020; Deng et al. 2024). However, some volatile components cannot be properly identified due to the lack of sufficient GC‐IMS database. Gas chromatography–mass spectrometry (GC–MS) coupled with solid‐phase microextraction (SPME) is also widely used for volatile flavor compounds analysis. W GC–MS offers greater capabilities on chemical identification by using comprehensive GC–MS databases when comparing to IMS technique. As a result, combining GC‐IMS with SPME‐GC–MS analysis will offer a straightforward analysis strategy to have thorough understanding of the volatile components for samples in food science (Wu et al. 2022; Gu et al. 2021).

In this study, GC‐IMS and SPME‐GC–MS were both employed to investigate fenugreek tincture samples from various manufacturers. The key flavor components of fenugreek tincture were determined by descriptive sensory analysis and the relationship between key flavor components and their aroma contributions was explored. This study will offer a theoretical analytical framework for future investigations of sensory quality control and extraction procedure improvement of fenugreek tincture products.

## 2 Materials and Methods

### 2.1 Materials, Reagents, and Instruments

**FIGURE 1:**
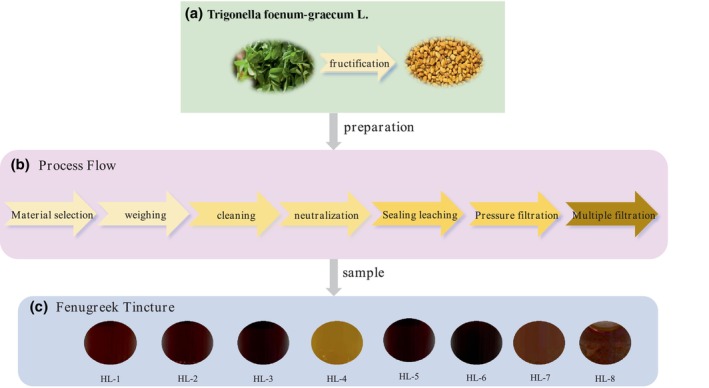
The preparation process and appearance colors of fenugreek tincture samples.

Fenugreek tinctures from eight different manufacturers are provided by Courtesy of Zhengzhou Tobacco Research Institute and the general production process of these samples are shown in Figure 1 (Sun, Zhang, and Li 2018a, 2018b).

Phenylethyl acetate (Standard, ≥ 99%, Fluka, Germany)；Sodium Chloride (AR, Sinopharm Chemical Reagent Co. Ltd); C7 ~ C40 n‐alkane mixed standard (1000 mg/L dissolved in n‐hexane, Shanghai Anpu Experimental Technology Co. Ltd) were used in this study.

7890B/5977 Gas chromatography‐mass spectrometer with Agilent MassHunter Workstation(Agilent, USA); Gas phase ion mobility spectrometer FlavourSpec (G.A.S., German); DF‐101S Collector type thermostatic heating magnetic stirrer (Gongyi Yuhua Instrument Co. Ltd); Divinylbenzene/carboxy/dimethicone (50/30 μm, DVB/CAR/PDMS) Extraction tip, SPME injection handle (Supelco Inc., USA) were used.

### 2.2 Method

#### 2.2.1 GC‐IMS Analysis

Fenugreek tincture sample of 1.0 g was placed in a 20 mL headspace bottle and incubated at 80°C for 10 min at 500 r/min. Injection temperature was set to 80°C and the injection volume was 500 μL. Measurements of each sample were carried out in triplicates. N_2_ (purity ≥ 99.999%) was used as carrier gas and the flow rate was set to 2 mL/min during 0–2 min, 2–10 mL/min during 2–10 min, 10–100 mL/min during 10–20 min, and 100 mL/min during 20–40 min. Inlet temperature was set to 75°C. GC column was using MXT‐5 (15 m × 0.53 mm, 1.0 μm) with temperature of 60°C. The drift tube length was 53 mm with temperature set to 45°C. The drift gas was N_2_ (purity ≥ 99.999%) with a flow rate of 150 mL/min. Tritium source was used as the radioactive source and the ionization mode was set to positive.

#### 2.2.2 SPME‐GC–MS Analysis

Fenugreek tincture sample of 1.0 g was combined with 0.5 g NaCl and 50 μL of 1.036 mg/mL internal standard n‐heptadecane inside a 20 mL headspace vial (PTFE pad). The vial was then tightly sealed and shaken well before incubating at 65°C for 30 min. The aged divinylbenzene/carboxy/polydimethylsiloxane (50/30 μm, DVB/CAR/PDMS) fiber was quickly poked into the vial for SPME thermal adsorption extraction with 30 min duration. After extraction process, the fiber was removed and placed into the 230°C injection inlet for 10 min. GC–MS analysis was then performed. Measurements of each sample were carried out in triplicates. DB‐5MS column (30 m × 0.25 mm, 0.25 μm) was used. Split‐less injection was employed with the temperature of injection inlet set to 250°C. Helium (≥ 99.999%) was used as the carrier gas and flow rate was 1 mL/min. The temperature programing was set as below: 40°C–250°C at 3°C/min and then kept at 250°C for 5 min. The ion source and quadrupole temperature were set to 150°C and 230°C, respectively and the electron energy was kept at 70 eV. Scan Mode was employed with a mass range of 30–450 amu. The solvent delay was set to 3 min.

#### 2.2.3 Identification and Quantification

The GC–MS results were qualitatively analyzed by matching the components over 85% in NIST 20 and FLAVOR2 database to calculate the Retention Index (RI) (Louw, Kandjengo, and Knott 2017). The RI results was then compared to the literature (webbook.nist.gov, www.flavornet.org, and standard mass database). The identification of the analytes are determined when the absolute RI value difference is within 20 from the database.

The internal standard method was used for quantification. The relative mass concentrations of each component are calculated according to equation (1):

(1) $$C_{i}=\frac{A_{i}}{A_{s}\times V}\times \mathrm{Ws}$$

where *C*_*i*_ is the concentration of the analyte (μg/mL); *A*_*i*_ and *A*_*s*_ are the peak areas of the analyte and internal standard, respectively; *V* is the volume of the sample solution (mL); *W*_*s*_ is the weight of the internal standard (μg). In this work, the correction factor of each analyte is assumed to be 1.

#### 2.2.4 Aroma Evaluation

DSA (Descriptive Sensory Analysis) was employed to evaluate the eight fenugreek tincture samples. The evaluation team consist of seven experienced members including five men and two women, aged between 25 and 45 years old. Prior to the evaluation, the whole team was trained according to the ISO 8589 (2007) protocol. Then the members were asked to describe the sensory properties of the sample solution as many as possible. The final evaluation results were comprehensively sort out. Resin, liquorice, burnt, alcohol, fresh, sweet, ointment, baked, woody, herb, bean, green, spicy aroma, fruit, floral, creamy, cocoa, and sour aromas were selected to evaluate the sample solution. The aroma intensity was scored on a 10‐point scale (0 for no aroma and 10 for the strongest aroma). Each sample was evaluated in triplicates.

OAV evaluation determines the contributions of each component to the overall flavor (Gemert 2015, 2018; He et al. 2020). The OAV of each component was calculated based on the ratio of its concentration over odor threshold from the aqueous solution. Compounds with OAV > 1 are considered as key flavor components, which contribute to the aroma formation of fenugreek tincture. OVA can be calculated by Equation (2):

(2) $$\mathrm{OAV}=\frac{C}{T}$$

where *C* is the concentration of each component (μg/g); *T* is the odor threshold of each component (mg/kg).

#### 2.2.5 Data Analysis

Mass spectral raw data was deconvoluted and processed using Agilent MassHunter Unknowns Analysis B10.1 software. Results were exported to CEF format files, and then imported into Agilent MassHunter Mass Profiler (MPP) 15.1 software for peak identification, peak alignment, filtering, and other pretreatments to obtain high‐quality data matrices. The GC‐IMS results were analyzed by VOCal software and identification was conducted through built‐in NIST and IMS databases. The Gallery Plot plug‐in and the Reporter plug‐in were used to plot a 2D top view and fingerprint map for volatile components, respectively. SIMCA‐P 14.1 software was used to centralize and normalize the data and principal component analysis (PCA) was then conducted. Orthogonal partial least squares discriminant analysis (OPLS‐DA) and variable importance in the projection (VIP) were plotted using the metaboanalyst (http://www.metaboanalyst.ca) to screen out components that were significantly different among groups. SPSS 26.0 software was used for one‐way ANOVA. ChiPlot (http://www.chiplot.online) was used to plot clustered (circles) and intergroup correlation clustered heatmaps, and the mapping was generated by Origin 2022 software.

## 3 Results and Discussion

### 3.1 Descriptive Sensory Analysis

**FIGURE 2:**
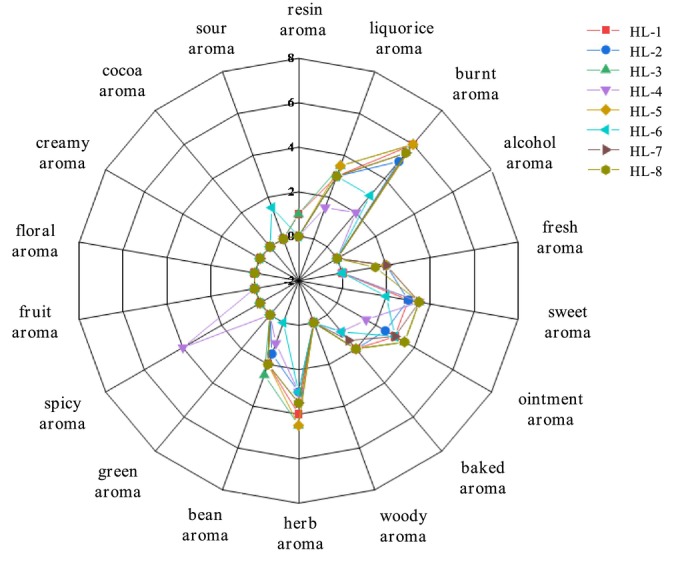
Radar chart of aroma attribute of different Fenugreek Tincture samples.

Samples of fenugreek tincture from various manufacturers were scored by DSA shown in Figure 2, and aroma differences were found in different samples. HL‐1, HL‐2, HL‐3, and HL‐5 have similar aroma properties which are mainly burnt, herbal, hay, sweet, and creamy aroma, with slightly different auxiliary aroma. For HL‐4, spicy aroma is predominated with strong sweet and herbal aroma, while burnt, hay, and creamy aroma are relatively weak. The main aroma of HL‐6 are burnt, herbal, hay, and ointment aroma. The most prominent aroma of HL‐7 and HL‐8 are burnt and sweet aroma, but their auxiliary aromas are different. The results show that the eight fenugreek tincture samples have a complex scent with burnt and herbal aroma as the prominent contributors with light sweet, hay, and creamy aroma. Furthermore, the scents of baked, fresh, bean, resin, and sour aroma are relatively weak.

### 3.2 Analysis of Volatile Components in Fenugreek Tincture Based on GC‐IMS

#### 3.2.1 Qualitative Analysis and Two‐Dimensional Spectrum Analysis of Volatile Components

GC‐IMS built‐in database was employed to evaluate the volatile components differences in the eight fenugreek tincture samples from various manufacturers. There were 37 volatile components that could be identified from all the samples including three components in the form of monomers or dimers. Thus, a total number of 34 volatile components were identified as shown in Table 1. All the identified components had carbon chains lengths between C_3_ and C_10_ and can be classified into eight categories, including alcohols (6), olefins (2), aldehydes (8), ketones (5), esters (10), pyridines (1), pyrazines (1), and pyrroles (1). Pyridine, ethyl formate, and N‐amyl alcohol exist as both monomers and dimers in the samples. The primary volatile components of fenugreek tincture are esters, aldehydes, and alcohols, which accounts for 65.26% of the total aroma components. This result is consistent with Su's work, which found that the primary volatile constituents in fenugreek tincture are esters (Su et al. 2012).

**TABLE 1:** GC‐IMS identification of volatile components and their relative content in different Fenugreek Tincture samples.

#	Compound	Retention index	Relative content/%							
			HL‐1	HL‐2	HL‐3	HL‐4	HL‐5	HL‐6	HL‐7	HL‐8
1	5‐Methyl‐2‐furfural	1585.2	0.27	0.28	0.19	0.30	0.22	0.55	0.27	0.29
2	Benzaldehyde	1513.2	29.95	30.65	21.55	34.27	25.18	10.21	30.99	30.45
3	Citronellal	1505.9	0.38	0.19	0.13	0.22	0.15	1.92	0.21	0.25
4	2,6‐Dimethyl‐pyrazine	1348.1	0.64	0.65	0.45	0.66	0.52	11.82	1.13	0.70
5	1‐Pentanol‐M	1257.2	0.17	0.15	0.10	0.15	0.10	0.39	0.09	0.10
6	1‐Pentanol‐D	1257.2	0.15	0.15	0.10	0.17	0.11	0.58	0.15	0.15
7	3‐Hydroxy‐2‐butanone	1257.2	0.17	0.12	0.11	0.12	0.14	1.24	0.20	0.18
8	Gamma‐Terpinene	1240.1	0.22	0.10	0.09	0.14	0.21	0.64	0.14	0.12
9	Butanoic acid butyl ester	1213.9	0.28	0.13	0.69	0.24	0.13	1.54	0.19	0.17
10	(E)‐2‐hexenal	1215.2	0.21	0.18	0.15	0.22	0.15	1.29	0.19	0.20
11	2‐Methylbutanol	1206.6	0.10	0.32	0.12	0.10	0.34	2.65	0.38	0.34
12	3‐Methyl‐2‐butenal	1188.8	0.14	0.12	0.10	0.12	0.33	1.51	0.13	0.13
13	2‐Heptanone	1189.3	0.79	0.60	0.29	0.18	2.25	1.23	1.07	0.63
14	4‐Methyl‐2‐pentanol	1170.9	0.03	0.05	0.04	0.02	0.03	0.31	0.03	0.08
15	Butyl propanoate	1150.8	1.90	0.17	0.10	0.16	9.96	1.95	0.21	0.21
16	Propyl butyrate	1143.4	0.82	0.52	0.26	0.14	0.95	1.65	0.69	0.87
17	2‐Methylpropyl butanoate	1138.8	15.61	18.26	12.09	20.13	14.62	7.83	17.96	18.85
18	(E)‐2‐pentenal	1114.1	0.13	0.10	0.27	0.08	0.09	1.29	0.11	0.34
19	2‐Methyl‐1‐propanol	1102.1	0.13	0.12	0.71	0.15	0.10	4.62	0.11	0.14
20	2‐Butanol	1033.8	0.39	0.15	0.17	0.18	0.46	1.76	0.36	0.20
21	2‐Methylpropyl 2‐methylpropanoate	1071.5	2.74	3.85	1.56	2.80	1.85	3.13	3.81	3.88
22	2‐Hexanone	1061.2	0.05	0.03	0.04	0.05	0.04	0.71	0.04	0.04
23	Pyrrolidine	988.8	0.06	0.05	0.04	0.07	0.04	0.98	0.06	0.06
24	Acetic acid propyl ester	990	0.08	0.08	0.08	0.08	0.14	2.96	0.20	0.22
25	Ethyl propanoate	970.3	0.27	0.20	0.53	0.23	0.18	0.65	0.12	0.14
26	1‐Propanol	1029.9	1.45	3.30	3.83	2.52	4.26	0.54	3.09	4.15
27	2‐Butanone	919.6	1.07	0.32	0.24	0.18	0.37	0.78	0.85	0.36
28	Acetic acid ethyl ester	882	9.94	11.85	6.76	7.62	8.39	4.13	11.47	12.10
29	Butanal	864.7	2.42	1.47	1.17	1.82	1.34	1.61	1.18	1.52
30	Propanal	850.4	0.92	0.26	0.18	0.33	0.25	0.60	0.72	0.44
31	Ethyl formate‐M	798.5	1.39	0.90	1.06	1.51	1.85	0.53	1.70	0.85
32	Ethyl formate‐D	799.7	0.59	0.60	0.20	0.26	1.36	1.14	0.53	0.60
33	Alpha‐Phellandrene	1152.7	3.24	1.65	0.87	1.57	1.31	0.54	1.16	1.21
34	2‐Methylbutyl acetate	1119.9	1.81	1.16	1.37	0.71	1.41	0.23	1.09	1.14
35	Pyridine‐M	1189	0.23	0.48	2.29	0.60	0.48	0.37	0.57	0.54
36	Pyridine‐D	1189	0.25	0.30	1.55	0.24	0.31	0.14	0.21	0.21
37	4‐Methyl‐3‐penten‐2‐one	1117.6	0.43	0.54	1.15	0.28	0.67	0.41	0.58	0.61

**FIGURE 3:**
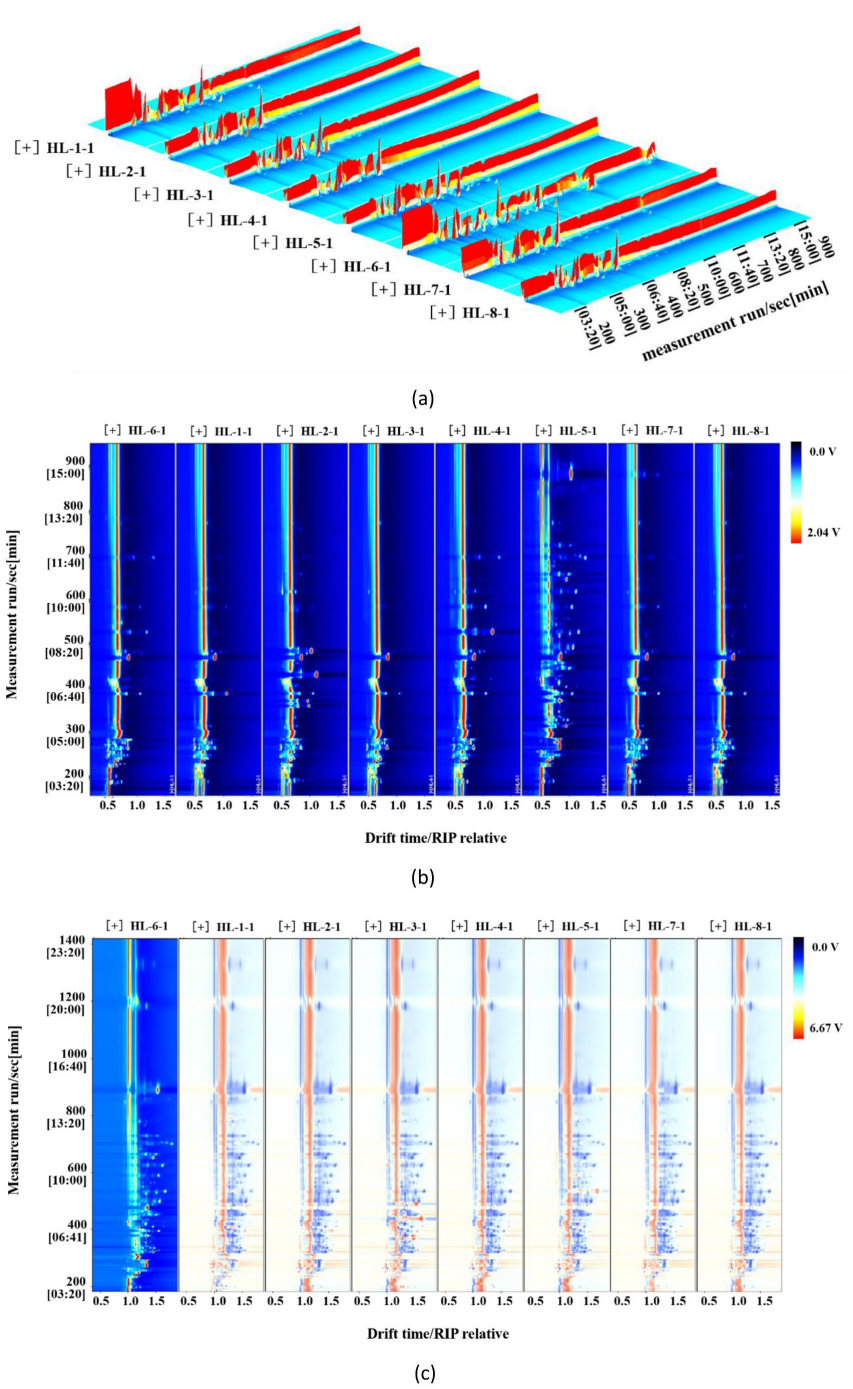
GC‐IMS spectra of different fenugreek tincture samples. (a) 3D view of GC‐IMS; (b) 2D view of GC‐IMS; (c) difference comparison chart.

The 3D spectra generated from the GC‐IMS analysis results is shown in Figure 3a with retention time shown in *y*‐axis, peak intensity shown in *z*‐axis, and ion migration time listed in *x*‐axis. It was found that the response of volatile components varies in different fenugreek tincture samples. Dimensionality reduction was processed for the 3D spectra to generate the 2D spectra, which is shown in Figure 3b, to get a more direct comparison. The red vertical line at abscissa 1.0 in Figure 3b represents the reactive ion peak (RIP). Normally, every dot on either side of the RIP represents one volatile component, however, some components with strong proton affinity may have more represented dots such as monomers, dimers, and trimers (Li et al. 2020). The number and color of the dots are representing the species and concentrations of the volatile components in the samples, respectively. More number of dots means more volatile species while the darker color represents higher concentration of the analyte. This result can provide a direct visual comparison of how the samples differ in terms of composition and concentration. Most volatile components have a retention time of 200–500 s, while some other components have a retention time of 550–750 s due to their low polarity, which results in more retention behavior in a non‐polar column (Chen et al. 2018).

HL‐6 was found to have significantly more volatile components comparing to the other samples. In order to compare the differences between samples more clearly, the differential comparison mode was employed to generate a 2D differential spectra (Figure 3c) by subtracting HL‐6 GC‐IMS spectrum from the remaining seven fenugreek tinctures spectra. The color of the spectra background was set to white. The red color represents a higher concentration of the component compared to the reference sample, while the blue color represents a lower concentration. The color and position of all the dots visually reflect the differences in volatile components between HL‐6 and other samples (Chen et al. 2018). All the other seven samples have substantially lower volatile component concentrations than HL‐6 which are depicted in blue dots, excluding benzaldehyde, trans‐2‐pentenal, methyl ethyl ketone, n‐butyraldehyde, ethyl formate‐dimer, hydrargyrum, 2‐methylbutyl acetate, and pyridine‐dimer. It suggests that fenugreek tinctures from various manufacturers clearly differ in terms of volatile component concentration and composition which may lead to a wide range of flavors.

#### 3.2.2 GC‐IMS Fingerprint Spectra Analysis

**FIGURE 4:**
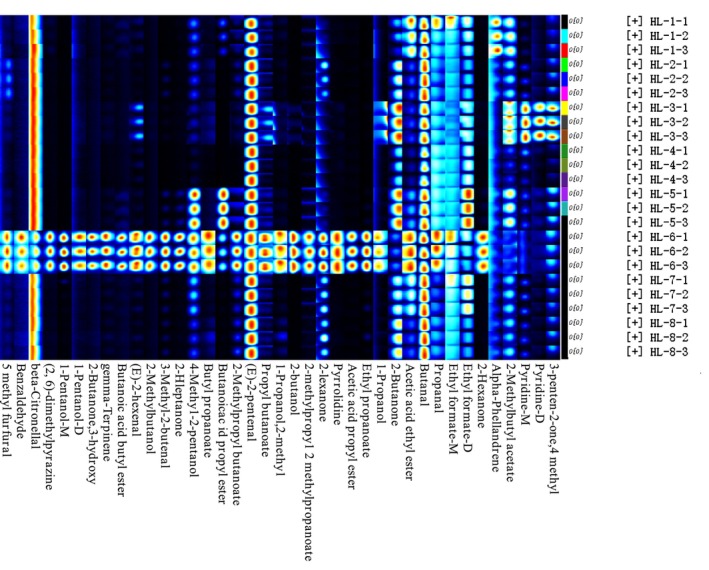
GC‐IMS fingerprint of volatile compounds in different fenugreek tincture samples.

The fingerprint spectra of the volatile components (shown in Figure 4) were generated by Gallery Plot mode to better evaluate the concentration differences of volatile components in fenugreek tincture samples. The response of the same volatile component in different samples are represented in each column, while the response collected from the same sample are represented in each row. The brightness of the response increases with the concentration of the component. This figure provides a direct visual comparison of the volatile components content from different samples. By longitudinal comparison, the different volatile components show certain patterns. When combined with the data listed in Table 1, it is clearly to see the fenugreek tincture samples shared a common flavor region and their characteristic peak regions as well. Higher concentrations of pyridine, n‐propyl acetate, n‐butyraldehyde, 2‐methylbutyl acetate, and trans‐2‐pentenal were found in HL‐1 while higher concentrations of methyl ethyl ketone, trans‐2‐pentenal, and n‐butyraldehyde were found in HL‐2. Greater concentrations of 2‐methylbutyl acetate, n‐butyraldehyde, trans‐2‐pentenal, methyl ethyl ketone, pyridine, and isopropylacetone were observed in HL‐3. Higher concentrations of n‐butyraldehyde, ethyl formate, and trans‐2‐pentenal components were found in HL‐4. Higher concentrations of methyl ethyl ketone, propyl butyrate, trans‐2‐pentenal, n‐butanal, and ethyl formate were detected in HL‐5. Higher concentrations of n‐butyraldehyde and trans‐2‐pentenal were found in HL‐7 and HL‐8. HL‐6 has a high concentration of 29 constituents, including 5‐methylfurfural, butyl propionate, and n‐propyl acetate, which has more species and substantially higher concentrations comparing to the rest fenugreek tincture samples. In comparison to the remaining samples, HL‐3, HL‐5, and HL‐6 had more volatile components in general. Trans‐2‐pentenal and n‐butyraldehyde were found to be present in all the samples.

### 3.3 Analysis of Volatile Components in Fenugreek Tincture Based on SPME‐GC–MS

Identification of the volatile components in fenugreek tincture samples obtained from various manufacturers were conducted by SPME‐GC–MS and the findings are listed in Table 2. A total number of 114 volatile compounds were found in eight fenugreek tincture samples, which can be mainly classified into ten categories: esters and lactones (47), furans (6), azepines (7), alcohols (7), hydrocarbons (8), phenols (4), aldehydes (12), ketones (7), acids and anhydrides (12), and other species (4). The content of volatile components varied greatly in samples from different manufacturers, which may be closely related to the production conditions differences.

**TABLE 2:** GC–MS analysis and identification of volatile compounds in different Fenugreek Tincture samples (*n* = 3).

**#** ^a^	Compound	RI^b^	Relative mass^c^/(μg/g)								Qualitative approach^d^
			HL‐1	HL‐2	HL‐3	HL‐4	HL‐5	HL‐6	HL‐7	HL‐8	
A1	Pyridine	772	n.d.^e^	n.d.	58.57 ± 3.92	n.d.	1.04 ± 0.05	n.d.	n.d.	1.08 ± 0.11	RI, MS
A2	2‐Methyl‐pyrazine	836	n.d.	6.19 ± 0.87	76.14 ± 2.92	n.d.	11.71 ± 1.46	n.d.	n.d.	2.49 ± 0.34	RI, MS
A3	2,6‐Dimethyl‐pyrazine	917	n.d.	0.57 ± 0.02	n.d.	n.d.	2.16 ± 0.07	n.d.	n.d.	n.d.	RI, MS
A4	Trimethyl‐pyrazine	1003	n.d.	4.05 ± 0.16	25.58 ± 1.07	0.97 ± 0.02	8.79 ± 0.27	0.19 ± 0.01	n.d.	1.46 ± 0.18	RI, MS
A5	3‐Ethyl‐2,5‐dimethyl‐pyrazine	1077	n.d.	n.d.	17.56 ± 1.55	n.d.	11.82 ± 0.98	0.67 ± 0.07	n.d.	1.07 ± 0.03	RI, MS
A6	2,6‐Diethyl‐pyrazine	1077	n.d.	1.19 ± 0.09	1.24 ± 0.11	n.d.	n.d.	0.14 ± 0.01	n.d.	0.02 ± 0.00	RI, MS
A7	2,5‐Dimethyl‐pyrazine	917	n.d.	14.59 ± 1.25	73.97 ± 2.89	2.23 ± 0.18	38.56 ± 1.67	n.d.	7.47 ± 0.45	6.73 ± 0.27	RI, MS
B1	1,2‐Propanediol	758	n.d.	n.d.	n.d.	n.d.	n.d.	n.d.	n.d.	40.95 ± 1.79	RI, MS
B2	Propylene Glycol	779	n.d.	638.59 ± 7.29	n.d.	n.d.	n.d.	n.d.	n.d.	168.29 ± 3.08	RI, MS
B3	1‐Hexanol	876	n.d.	n.d.	n.d.	n.d.	n.d.	0.02 ± 0.00	n.d.	n.d.	RI, MS
B4	Phenylethyl Alcohol	1117	n.d.	n.d.	n.d.	4.01 ± 0.16	n.d.	0.73 ± 0.04	n.d.	n.d.	RI, MS
B5	DL‐Menthol	1181	n.d.	n.d.	n.d.	n.d.	n.d.	0.04 ± 0.01	0.27 ± 0.03	0.26 ± 0.01	RI, MS
B6	Cadinol	1618	n.d.	n.d.	n.d.	n.d.	n.d.	0.21 ± 0.03	n.d.	n.d.	RI, MS
B7	(1S,4R,4aS,8aR)‐1,3,4,5,6,8a‐hexahydro‐4,7‐dimethyl‐1‐(1‐methylethyl)‐4a(2H)‐Naphthalenol	1619	n.d.	n.d.	n.d.	1.93 ± 0.07	2.50 ± 0.12	1.19 ± 0.09	n.d.	n.d.	RI, MS
C1	Phenol	977	n.d.	n.d.	n.d.	n.d.	n.d.	0.44 ± 0.01	n.d.	n.d.	RI, MS
C2	Maltol	1112	n.d.	n.d.	n.d.	n.d.	n.d.	0.11 ± 0.02	n.d.	n.d.	RI, MS
C3	4‐Ethyl‐phenol	1163	n.d.	n.d.	n.d.	n.d.	n.d.	0.19 ± 0.02	n.d.	n.d.	RI, MS
C4	Butylated Hydroxytoluene	1503	n.d.	n.d.	n.d.	n.d.	n.d.	0.13 ± 0.01	n.d.	n.d.	RI, MS
D1	2,3‐Dihydro‐4‐methyl‐furan	760	n.d.	n.d.	2.15 ± 0.06	n.d.	n.d.	n.d.	36.51 ± 0.55	n.d.	RI、MS
D2	2‐Acetylfuran	911	11.39 ± 0.59	n.d.	n.d.	n.d.	3.57 ± 0.20	0.03 ± 0.00	n.d.	n.d.	RI, MS
D3	2‐Pentyl‐furan	991	n.d.	n.d.	n.d.	n.d.	n.d.	0.19 ± 0.01	n.d.	n.d.	RI, MS
D4	3‐Hydroxy‐4,5‐dimethyl‐2 (5H)‐furanone	1095	2.05 ± 0.08	1.17 ± 0.04	n.d.	2.92 ± 0.03	6.81 ± 0.16	0.87 ± 0.02	1.69 ± 0.09	1.13 ± 0.07	RI, MS
D5	Ethylfuranone	1139	n.d.	n.d.	n.d.	n.d.	n.d.	0.13 ± 0.05	n.d.	n.d.	RI, MS
D6	3‐Amino‐4,5‐dimethyl‐2 (5H)‐furanone	1234	2.58 ± 0.17	0.52 ± 0.03	0.50 ± 0.02	3.10 ± 0.01	5.98 ± 0.13	n.d.	n.d.	0.43 ± 0.03	RI, MS
E1	1‐Methoxy‐4‐propyl‐benzene	1208	n.d.	n.d.	n.d.	n.d.	n.d.	0.01 ± 0.00	n.d.	n.d.	RI, MS
E2	Fluorene	1594	n.d.	n.d.	n.d.	n.d.	1.01 ± 0.02	n.d.	n.d.	n.d.	RI, MS
E3	4,5‐Dimethyl‐isothiazole	973	n.d.	n.d.	3.38 ± 0.07	n.d.	n.d.	n.d.	n.d.	n.d.	RI, MS
E4	3,5‐Dimethylpyrazole‐1‐carboxamide	1324	n.d.	n.d.	n.d.	n.d.	n.d.	0.03 ± 0.00	n.d.	n.d.	RI, MS
F1	2‐Methyl‐2‐butenal	766	121.61 ± 2.70	n.d.	100.56 ± 1.52	88.46 ± 1.38	167.59 ± 3.75	1.37 ± 0.08	63.28 ± 1.33	40.85 ± 1.21	RI, MS
F2	3‐Furaldehyde	840	n.d.	n.d.	n.d.	n.d.	7.34 ± 0.16	5.17 ± 0.20	n.d.	n.d.	RI, MS
F3	Furfural	836	322.72 ± 4.87	35.48 ± 2.08	35.21 ± 1.77	n.d.	4.64 ± 0.35	0.59 ± 0.04	10.31 ± 0.19	20.65 ± 0.55	RI, MS
F4	5‐Methyl‐2‐furancarboxaldehyde	963	12.46 ± 0.99	n.d.	n.d.	n.d.	n.d.	0.64 ± 0.15	n.d.	n.d.	RI, MS
F5	Benzaldehyde	965	15.09 ± 0.19	58.06 ± 1.01	8.91 ± 0.56	2.51 ± 0.11	16.29 ± 0.17	3.93 ± 0.08	6.29 ± 0.32	4.72 ± 0.26	RI, MS
F6	2‐Ethyl‐2‐hexenal	1007	n.d.	n.d.	n.d.	n.d.	n.d.	0.27 ± 0.03	n.d.	n.d.	RI, MS
F7	1‐Methyl‐1H‐pyrrole‐2‐carboxaldehyde	1007	n.d.	n.d.	4.88 ± 0.24	n.d.	n.d.	n.d.	n.d.	n.d.	RI, MS
F8	1H‐pyrrole‐2‐carboxaldehyde	1011	n.d.	n.d.	4.36 ± 0.18	n.d.	n.d.	n.d.	n.d.	n.d.	RI, MS
F9	2‐Propenylhydrazone‐propanal	1058	n.d.	n.d.	n.d.	n.d.	n.d.	0.07 ± 0.01	n.d.	n.d.	RI, MS
F10	Nonanal	1105	n.d.	n.d.	n.d.	n.d.	n.d.	0.10 ± 0.00	n.d.	n.d.	RI, MS
F11	3‐Ethyl‐benzaldehyde	1167	n.d.	n.d.	n.d.	n.d.	n.d.	0.09 ± 0.01	n.d.	n.d.	RI, MS
F12	Alpha.‐ethylidene‐benzeneacetaldehyde	1272	n.d.	n.d.	n.d.	n.d.	n.d.	0.08 ± 0.00	n.d.	n.d.	RI, MS
G1	Propanoic acid	743	n.d.	n.d.	n.d.	n.d.	3.28 ± 0.07	n.d.	n.d.	n.d.	RI, MS
G2	2‐Methyl‐butanoic acid	849	n.d.	n.d.	n.d.	n.d.	7.13 ± 0.15	n.d.	n.d.	0.45 ± 0.02	RI, MS
G3	Butanoic acid	797	n.d.	6.71 ± 0.55	n.d.	n.d.	n.d.	n.d.	n.d.	2.10 ± 0.07	RI, MS
G4	2,2‐Dimethyl‐butanoic acid	810	n.d.	n.d.	n.d.	n.d.	n.d.	n.d.	1.84 ± 0.28	n.d.	RI, MS
G5	Pentanoic acid	880	n.d.	n.d.	n.d.	n.d.	n.d.	0.08 ± 0.01	n.d.	1.39 ± 0.03	RI, MS
G6	2‐Methyl‐2‐butenoic acid	924	n.d.	n.d.	n.d.	n.d.	28.81 ± 1.66	0.19 ± 0.05	n.d.	n.d.	RI, MS
G7	Tiglic acid	926	n.d.	n.d.	n.d.	n.d.	16.69 ± 0.87	n.d.	n.d.	n.d.	RI, MS
G8	3‐Methyl‐pentanoic acid	980	n.d.	n.d.	n.d.	n.d.	n.d.	0.70 ± 0.01	n.d.	n.d.	RI, MS
G9	Hexanoic acid	982	n.d.	n.d.	n.d.	n.d.	n.d.	1.31 ± 0.04	n.d.	n.d.	RI, MS
G10	Heptanoic acid	1063	n.d.	n.d.	n.d.	n.d.	n.d.	0.17 ± 0.01	n.d.	n.d.	RI, MS
G11	n‐Decanoic acid	1357	n.d.	n.d.	n.d.	n.d.	n.d.	0.17 ± 0.02	n.d.	n.d.	RI, MS
G12	Acetic anhydride	716	n.d.	n.d.	n.d.	n.d.	n.d.	0.72 ± 0.08	n.d.	n.d.	RI, MS
H1	1,1‐Diethoxy‐ethane	702	56.79 ± 2.67	48.63 ± 1.89	16.96 ± 1.05	n.d.	76.39 ± 3.08	1.40	53.36 ± 0.66	46.09 ± 1.35	RI, MS
H2	1,1‐Diethoxy‐3‐methyl‐butane	949	n.d.	n.d.	n.d.	0.49 ± 0.03	0.28 ± 0.01	n.d.	n.d.	n.d.	RI, MS
H3	1,2‐Dipropenyl‐cyclobutane	1032	n.d.	n.d.	n.d.	0.34 ± 0.01	n.d.	n.d.	n.d.	n.d.	RI, MS
H4	D‐Limonene	1032	4.66 ± 0.21	10.23 ± 0.45	n.d.	10.89 ± 1.17	9.99 ± 0.57	n.d.	4.92 ± 0.03	n.d.	RI, MS
H5	1,1‐Diethoxy‐hexane	1087	n.d.	n.d.	n.d.	n.d.	n.d.	0.09 ± 0.01	n.d.	n.d.	RI, MS
H6	Bibenzyl	1527	n.d.	n.d.	n.d.	n.d.	n.d.	0.11 ± 0.03	n.d.	n.d.	RI, MS
H7	I‐Calamenene	1527	n.d.	n.d.	0.72 ± 0.02	n.d.	n.d.	n.d.	n.d.	n.d.	RI, MS
H8	Anethole	1289	n.d.	n.d.	n.d.	278.70 ± 6.89	n.d.	n.d.	n.d.	n.d.	RI, MS
I1	Acetoin	755	n.d.	n.d.	n.d.	3.59 ± 0.17	n.d.	n.d.	n.d.	n.d.	RI, MS
I2	Cyclopentanone	768	n.d.	n.d.	n.d.	n.d.	n.d.	0.82 ± 0.06	n.d.	n.d.	RI, MS
I3	Acetophenone	1070	n.d.	n.d.	n.d.	n.d.	n.d.	0.02 ± 0.00	n.d.	n.d.	RI, MS
I4	N‐Isopropyl‐4‐piperidone	1113	n.d.	n.d.	n.d.	n.d.	n.d.	0.08 ± 0.01	n.d.	n.d.	RI, MS
I5	(E)‐1‐(2,6,6‐trimethyl‐1,3‐cyclohexadien‐1‐yl)‐2‐Buten‐1‐one	1382	n.d.	n.d.	n.d.	n.d.	n.d.	0.02 ± 0.00	n.d.	n.d.	RI, MS
I6	(E)‐6,10‐dimethyl‐5,9‐undecadien‐2‐one	1446	n.d.	n.d.	n.d.	n.d.	n.d.	0.08 ± 0.01	n.d.	n.d.	RI, MS
I7	6,10,14‐Trimethyl‐2‐pentadecanone	1837	n.d.	n.d.	n.d.	n.d.	n.d.	0.19 ± 0.03	n.d.	n.d.	RI, MS
J1	(R)‐5,6,7,7a‐tetrahydro‐4,4,7a‐trimethyl‐2 (4H)‐benzofuranone	1537	n.d.	n.d.	n.d.	n.d.	n.d.	0.29 ± 0.02	n.d.	n.d.	RI, MS
J2	Butyl ester acetic acid	820	n.d.	n.d.	60.21 ± 1.15	n.d.	n.d.	n.d.	n.d.	n.d.	RI, MS
J3	Ethyl lactate	821	n.d.	n.d.	n.d.	n.d.	n.d.	1.09 ± 0.21	n.d.	n.d.	MS
J4	(E)‐2‐Butenoic acid ethyl ester	849	n.d.	n.d.	n.d.	n.d.	4.90 ± 0.66	n.d.	n.d.	n.d.	RI, MS
J5	Pentanoic acid ethyl ester	901	n.d.	n.d.	n.d.	n.d.	20.67 ± 1.09	n.d.	n.d.	n.d.	RI, MS
J6	Ethyl tiglate	938	n.d.	4.53 ± 0.07	2.87 ± 0.03	5.96 ± 0.26	17.67 ± 0.59	0.14 ± 0.02	6.57 ± 0.49	3.19 ± 0.29	RI, MS
J7	Hexanoic acid ethyl ester	998	2.77 ± 0.65	n.d.	n.d.	n.d.	12.85 ± 0.78	0.24 ± 0.02	n.d.	n.d.	RI, MS
J8	2‐Hydroxy‐4‐methyl‐, ethyl ester pentanoic acid	1056	n.d.	n.d.	n.d.	n.d.	n.d.	0.39 ± 0.04	n.d.	n.d.	RI, MS
J9	DL‐2‐hydroxycaproate ethyl	1058	n.d.	n.d.	n.d.	n.d.	n.d.	0.46 ± 0.01	n.d.	n.d.	RI, MS
J10	Heptanoic acid ethyl ester	1096	n.d.	n.d.	n.d.	n.d.	0.74 ± 0.03	n.d.	n.d.	n.d.	RI, MS
J11	Ethyl hydrogen succinate	1161	n.d.	n.d.	n.d.	n.d.	n.d.	0.16 ± 0.01	n.d.	n.d.	RI, MS
J12	Benzoic acid ethyl ester	1172	1.43 ± 0.20	n.d.	n.d.	n.d.	n.d.	0.04 ± 0.00	n.d.	n.d.	RI, MS
J13	Butanedioic acid diethyl ester	1172	n.d.	n.d.	n.d.	n.d.	n.d.	2.09 ± 0.11	n.d.	n.d.	RI, MS
J14	7‐Octenoic acid ethyl ester	1185	n.d.	n.d.	n.d.	n.d.	n.d.	0.05 ± 0.00	n.d.	n.d.	RI, MS
J15	Octanoic acid ethyl ester	1194	0.36 ± 0.03	n.d.	n.d.	n.d.	0.55 ± 0.01	0.17 ± 0.01	n.d.	n.d.	RI, MS
J16	Acetic acid octyl ester	1208	n.d.	n.d.	n.d.	n.d.	0.53 ± 0.02	0.06 ± 0.00	n.d.	n.d.	RI, MS
J17	Benzeneacetic acid ethyl ester	1243	n.d.	n.d.	n.d.	n.d.	n.d.	0.94 ± 0.05	n.d.	n.d.	RI, MS
J18	Pentanedioic acid diethyl ester	1276	n.d.	n.d.	n.d.	n.d.	n.d.	0.02 ± 0.00	n.d.	n.d.	RI, MS
J19	Benzenepropanoic acid ethyl ester	1349	n.d.	n.d.	n.d.	n.d.	n.d.	0.04 ± 0.00	n.d.	n.d.	RI, MS
J20	γ‐Nonolactone	1361	n.d.	n.d.	n.d.	n.d.	n.d.	0.19 ± 0.01	n.d.	n.d.	RI, MS
J21	Decanoic acid ethyl ester	1391	0.90 ± 0.05	n.d.	n.d.	n.d.	1.99 ± 0.33	0.18 ± 0.01	n.d.	n.d.	RI, MS
J22	Nonanoic acid ethyl ester	1399	n.d.	n.d.	n.d.	n.d.	n.d.	0.45 ± 0.02	n.d.	n.d.	RI, MS
J23	4‐Methoxy‐ acetate‐benzenemethanol	1417	n.d.	n.d.	n.d.	n.d.	0.61 ± 0.03	n.d.	n.d.	n.d.	RI, MS
J24	Ethyl 2‐hydroxy‐3‐phenylpropanoate	1441	n.d.	n.d.	n.d.	n.d.	n.d.	0.06 ± 0.01	n.d.	n.d.	RI, MS
J25	Diethyl pimelate	1579	n.d.	n.d.	n.d.	n.d.	n.d.	0.09 ± 0.00	n.d.	n.d.	RI, MS
J26	4‐Methoxyphenylacetic acid ethyl ester	1496	n.d.	n.d.	n.d.	n.d.	n.d.	0.37 ± 0.12	n.d.	n.d.	RI, MS
J27	9‐Oxo‐nonanoic acid ethyl ester	1501	n.d.	n.d.	n.d.	n.d.	n.d.	1.09 ± 0.07	n.d.	n.d.	RI, MS
J28	Diethyl suberate	1579	n.d.	n.d.	n.d.	n.d.	n.d.	0.21 ± 0.01	n.d.	n.d.	RI, MS
J29	Dodecanoic acid ethyl ester	1589	1.42 ± 0.04	n.d.	n.d.	n.d.	3.46 ± 0.16	0.61 ± 0.07	n.d.	n.d.	RI, MS
J30	Diethyl azelate	1679	n.d.	n.d.	n.d.	n.d.	n.d.	1.17 ± 0.02	n.d.	n.d.	RI, MS
J31	Ethyl tridecanoate	1689	n.d.	n.d.	n.d.	n.d.	n.d.	0.21 ± 0.01	n.d.	n.d.	RI, MS
J32	Benzyl Benzoate	1773	n.d.	n.d.	n.d.	n.d.	n.d.	n.d.	0.17 ± 0.02	n.d.	RI, MS
J33	Decanedioic acid diethyl ester	1779	n.d.	n.d.	n.d.	n.d.	n.d.	0.04 ± 0.00	n.d.	n.d.	RI, MS
J34	Tetradecanoic acid ethyl ester	1788	n.d.	n.d.	n.d.	n.d.	0.53 ± 0.04	1.62 ± 0.24	n.d.	n.d.	RI, MS
J35	(Z)‐Ethyl pentadec‐9‐enoate	1872	n.d.	n.d.	n.d.	n.d.	n.d.	0.39 ± 0.03	n.d.	n.d.	RI, MS
J36	Pentadecanoic acid ethyl ester	1888	n.d.	n.d.	n.d.	n.d.	n.d.	0.89 ± 0.20	n.d.	n.d.	RI, MS
J37	Dibutyl phthalate	1953	n.d.	n.d.	1.99 ± 0.32	1.31 ± 0.14	n.d.	n.d.	n.d.	n.d.	RI, MS
J38	9‐Hexadecenoate ethyl	1968	n.d.	n.d.	n.d.	n.d.	n.d.	0.24 ± 0.02	n.d.	n.d.	RI, MS
J39	Hexadecanoic acid ethyl ester	1988	14.69 ± 1.02	n.d.	n.d.	6.97 ± 0.29	26.06 ± 0.48	40.19 ± 3.62	n.d.	n.d.	RI, MS
J40	(Z)‐Ethyl heptadec‐9‐enoate	2066	n.d.	n.d.	n.d.	n.d.	n.d.	0.09 ± 0.01	n.d.	n.d.	RI, MS
J41	Heptadecanoic acid ethyl ester	2089	n.d.	n.d.	n.d.	n.d.	n.d.	0.86 ± 0.02	n.d.	n.d.	RI, MS
J42	Linoleic acid ethyl ester	2159	n.d.	n.d.	n.d.	1.26 ± 0.28	4.13 ± 0.55	11.38 ± 1.17	n.d.	n.d.	RI, MS
J43	Sulfurous acid, di(cyclohexylmethyl) ester	2164	0.83 ± 0.06	n.d.	n.d.	n.d.	n.d.	n.d.	n.d.	n.d.	RI, MS
J44	(E)‐9‐octadecenoic acid ethyl ester	2165	n.d.	n.d.	n.d.	n.d.	n.d.	3.20 ± 0.11	n.d.	n.d.	RI, MS
J45	9‐octadecenoic acid ethyl ester	2171	n.d.	n.d.	n.d.	0.83 ± 0.02	1.48 ± 0.23	1.15 ± 0.08	n.d.	n.d.	RI, MS
J46	Octadecanoic acid ethyl ester	2190	n.d.	n.d.	n.d.	n.d.	n.d.	1.76 ± 0.12	n.d.	n.d.	RI, MS
J47	2‐Hydroxy‐propanoic acid ethyl ester	826	n.d.	n.d.	n.d.	n.d.	n.d.	20.65 ± 0.52	n.d.	n.d.	RI, MS

^a^ A‐J stand for azacyclic, alcohol, phenol, furan, other, aldehyde, acid and anhydride, hydrocarbon, ketone, and ester and lactone in that order.

^b^ RI was calculated using DB‐5 ms column (30 m × 0.25 mm × 0.25 μm).

^c^ HL‐1 ~ HL‐8 denoted the eight different tinctures of fenugreek, and the data in the table were the mean relative mass of the samples of the respective groups.

^d^ RI represents the retention index identification; MS represents the mass spectrometry identification.

^e^ n.d. represents Not Detected.

**FIGURE 5:**
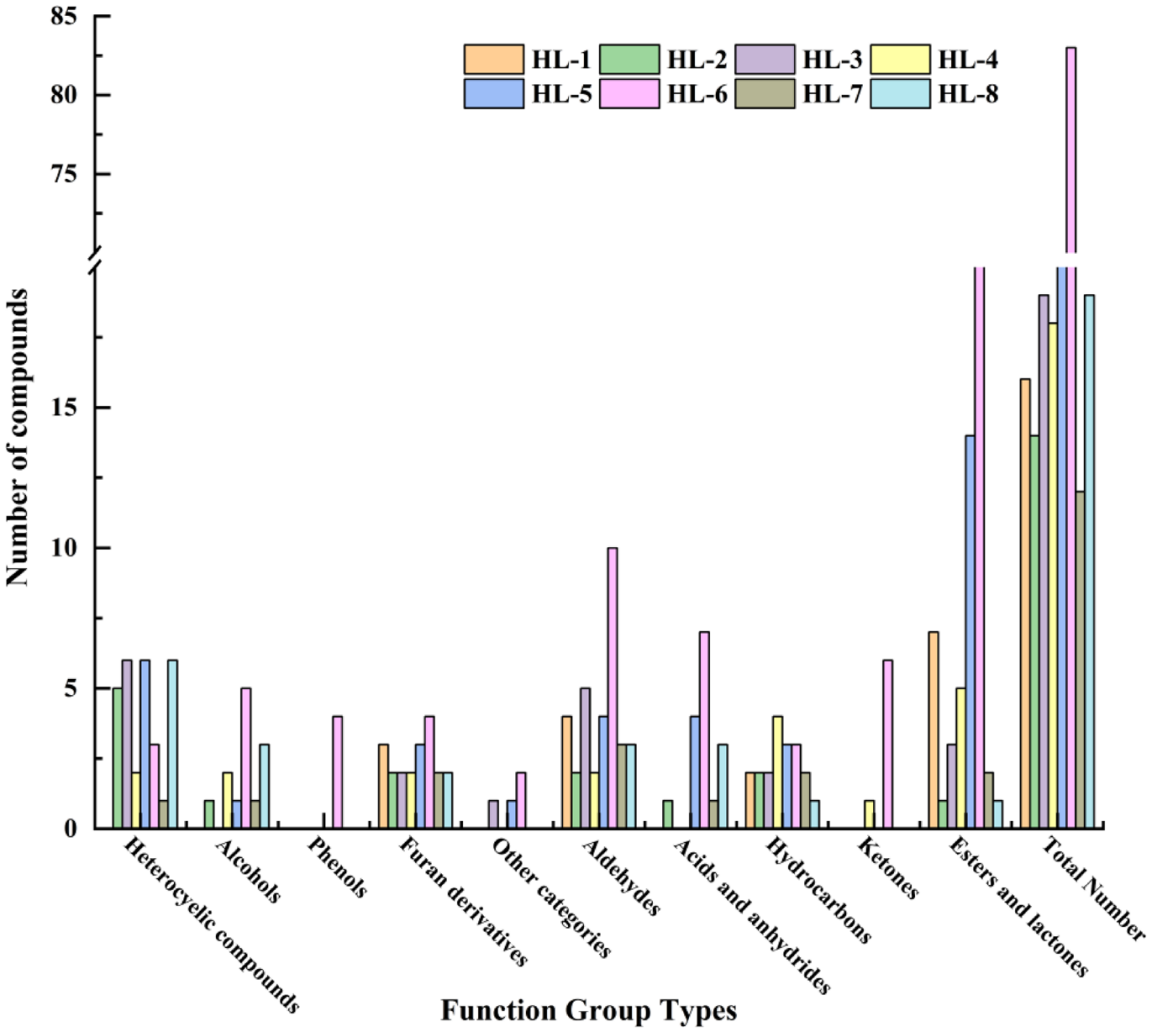
Number of volatile compound species in different fenugreek tincture samples.

**FIGURE 6:**
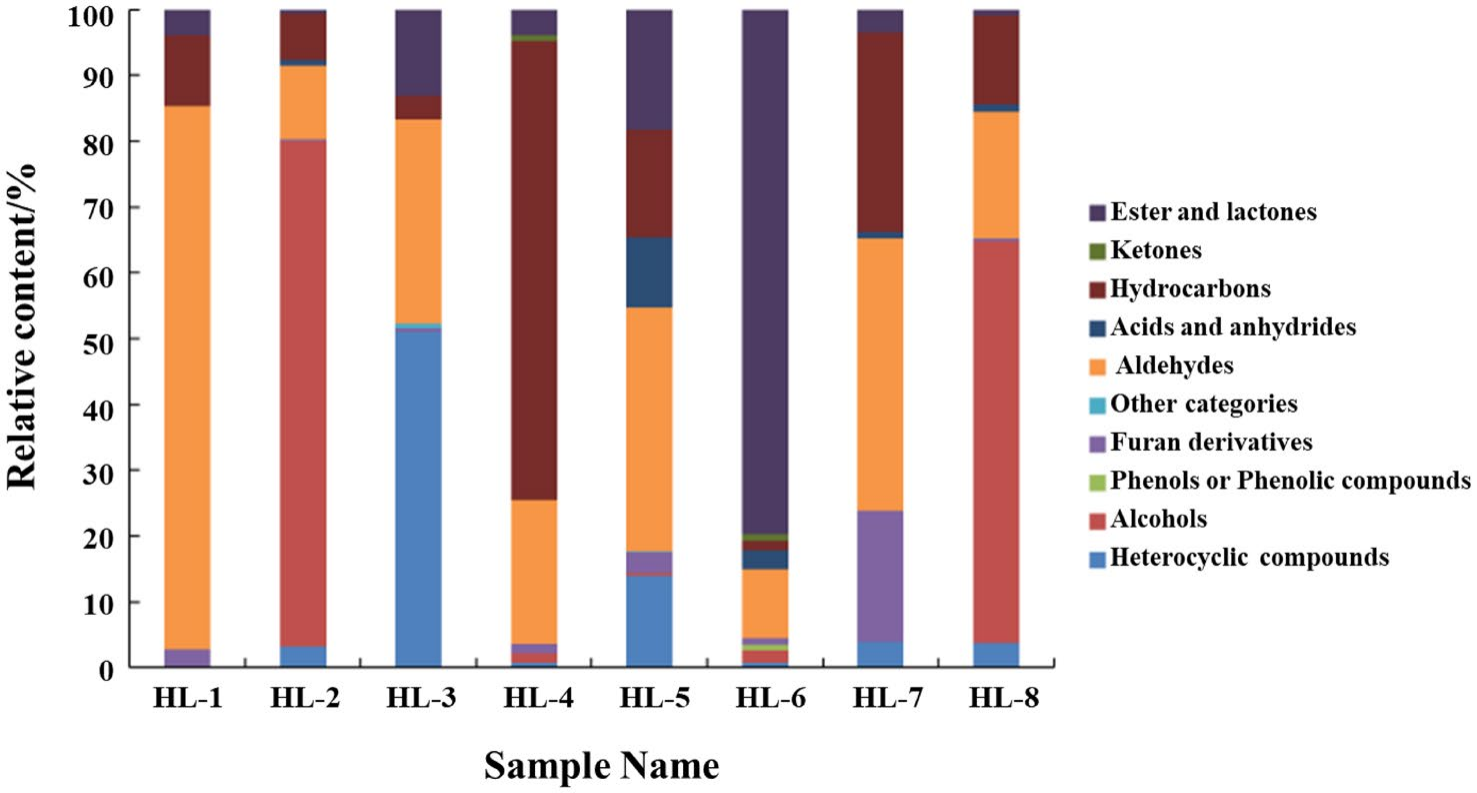
Relative content of different volatile compound species in different fenugreek tincture samples.

**FIGURE 7:**
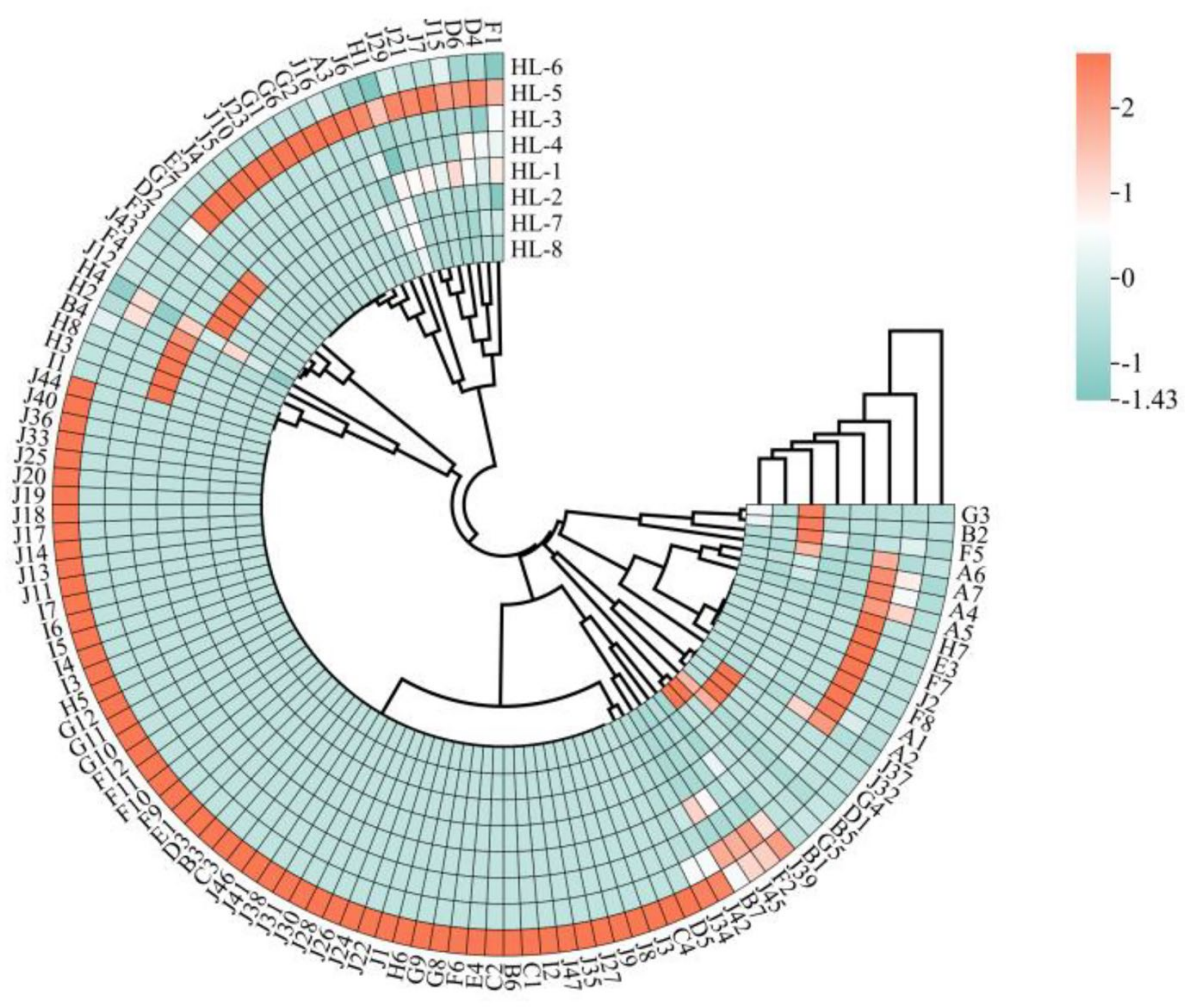
Clustering heat map of substance components in different fenugreek tincture samples.

The volatile components of the eight fenugreek tincture samples was analyzed and compared (shown in Figures 5 and 6). Overall, azepines, aldehydes and esters, and lactones account for the greatest number of components in all the samples. Then the species with high to low concentration follows as aldehyde hydrocarbons, alcohols, esters and lactones, and azepines. Several components have relative higher concentrations and more commonly exist in all the samples such as furfural (0 ~ 322.72 μg/g), 2‐methyl‐2‐butenal (0 ~ 167.59 μg/g), benzoin aldehyde (2.51 ~ 58.06 μg/g), 1,1‐diethoxyethane (0 ~ 76.39 μg/g), 2,5‐dimethyl pyrazine (0 ~ 73.97 μg/g), and ethyl tiglate (0 ~ 17.67 μg/g). The majority of these ingredients are aldehydes, which have pronounced burnt, baked, and fresh aroma. The outcomes aligned with the primary flavor profile of fenugreek tincture which was determined by previous sensory evaluation. The samples were also examined with a clustered heat map (Figure 6) to determine the variability of volatile components between samples and simplify the data set. The low to high content listed in Figure 7 are represented by colors ranging from green to red. HL‐3, HL‐5, and HL‐6 clustered largely into one group, while the other clustering group is formed by the remaining samples. The color bricks of HL‐3, HL‐5, and HL‐6 were generally darker than that of the other five samples, and HL‐6 varies largely when comparing to the other samples especially. The result largely agreed with the GC‐IMS fingerprint profiles of the eight samples shown in Figure 4. This implies that there are noticeable variations in the volatile components among the fenugreek tincture samples obtained from different manufacturers. It is worth noting that the volatile components identified by GC‐IMS were primarily alcohols, esters, and aldehydes, whereas the ones identified by GC–MS were primarily lactones, azepines, and aldehydes and esters. This may due to the different pretreatment, separation, enrichment, and detection techniques used in GC‐IMS and SPME‐GC–MS, which implies that the two techniques can be complementary to each other for volatile components identification (Chen et al. 2021). Aldehydes and esters were largely detected from both techniques, which may indicate that they play a significant role in fenugreek tincture flavor formation.

### 3.4 Analysis of the Characteristic Volatile Components of Fenugreek Tincture

#### 3.4.1 Principal Component Analysis of Volatile Components

**FIGURE 8:**
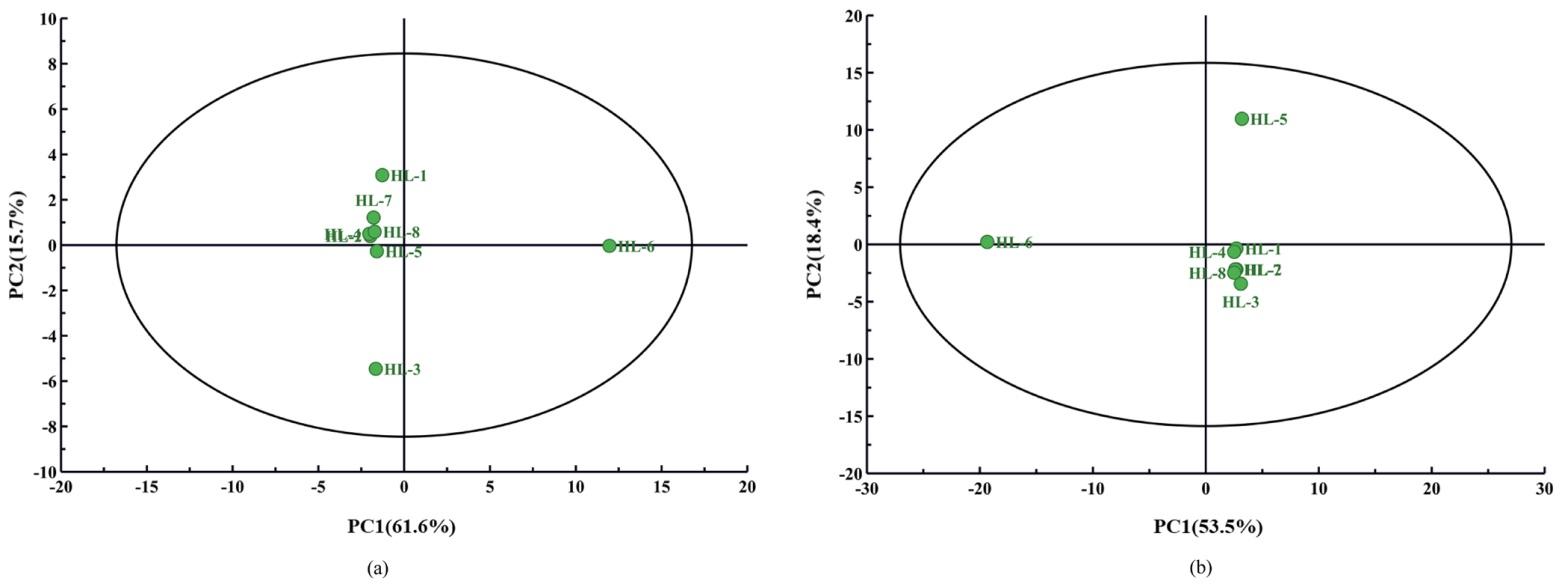
PCA plot of characteristic volatile components in different fenugreek tincture samples. (a) Chart of PCA scores for GC‐IMS; (b) Chart of PCA scores for SPME‐GC–MS.

Based on GC‐IMS and SPME‐GC–MS analysis results, PCA was employed to observe the distribution characteristics of multidimensional data, which are shown in Figure 8a,b, respectively. As shown in Figure 8a, the 2 PC (PC1 at 61.6% and PC2 at 15.7%) account for 77.3% of the variance in GC‐IMS analysis. This suggests that the great bulk of the reliable information in the original data can be represented by these 2 PC. All the samples fell inside the 95% confidence interval. In the primary component space, HL‐3 and HL‐6 are distributed in two comparatively separate regions. HL‐3 is mainly distributed on the negative semi‐axis of the second principal component while HL‐6 is mainly distributed on the positive semi‐axis of the first principal component. The remaining samples fell into a single category which indicates that their constituent parts are quite similar. The 2 PC (PC1 at 53.5% and PC2 at 18.4%) account for 71.9% of the variance in Figure 8b for SPME‐GC–MS analysis. This suggests that the great bulk of the reliable information in the original data can be represented by these 2 PC. All the samples fell inside the 95% confidence interval. In the primary component space, HL‐5 and HL‐6 are distributed in two comparatively separate regions. HL‐5 is predominantly distributed on the positive semi‐axis of the second principal component, while HL‐6 is predominantly distributed on the positive semi‐axis of the first principal component. The remaining samples fell into a single category which indicates that their constituent parts are quite similar. According to both PCA results, sample HL‐1, HL‐2, HL‐4, HL‐7, and HL‐8 can be grouped together, which indicates that their flavor profiles are similar. The performance of the remaining three samples was quite different. Especially, the distribution of HL‐6 differs significantly from that of the other samples. It can be found that HL‐6 has the darkest appearance color, followed by HL‐3 and HL‐5, where the other five samples have comparatively lighter colors. The corresponding relationship between color and component distribution is clearly seen when comparing the PCA distribution results. The appearance color of the samples is essentially the consequence of many combined processing parameters during the manufacturing of the fenugreek tincture and the volatile components are correspondingly linked to the appearance colors.

#### 3.4.2 Screening of Differential Volatility Components

**FIGURE 9:**
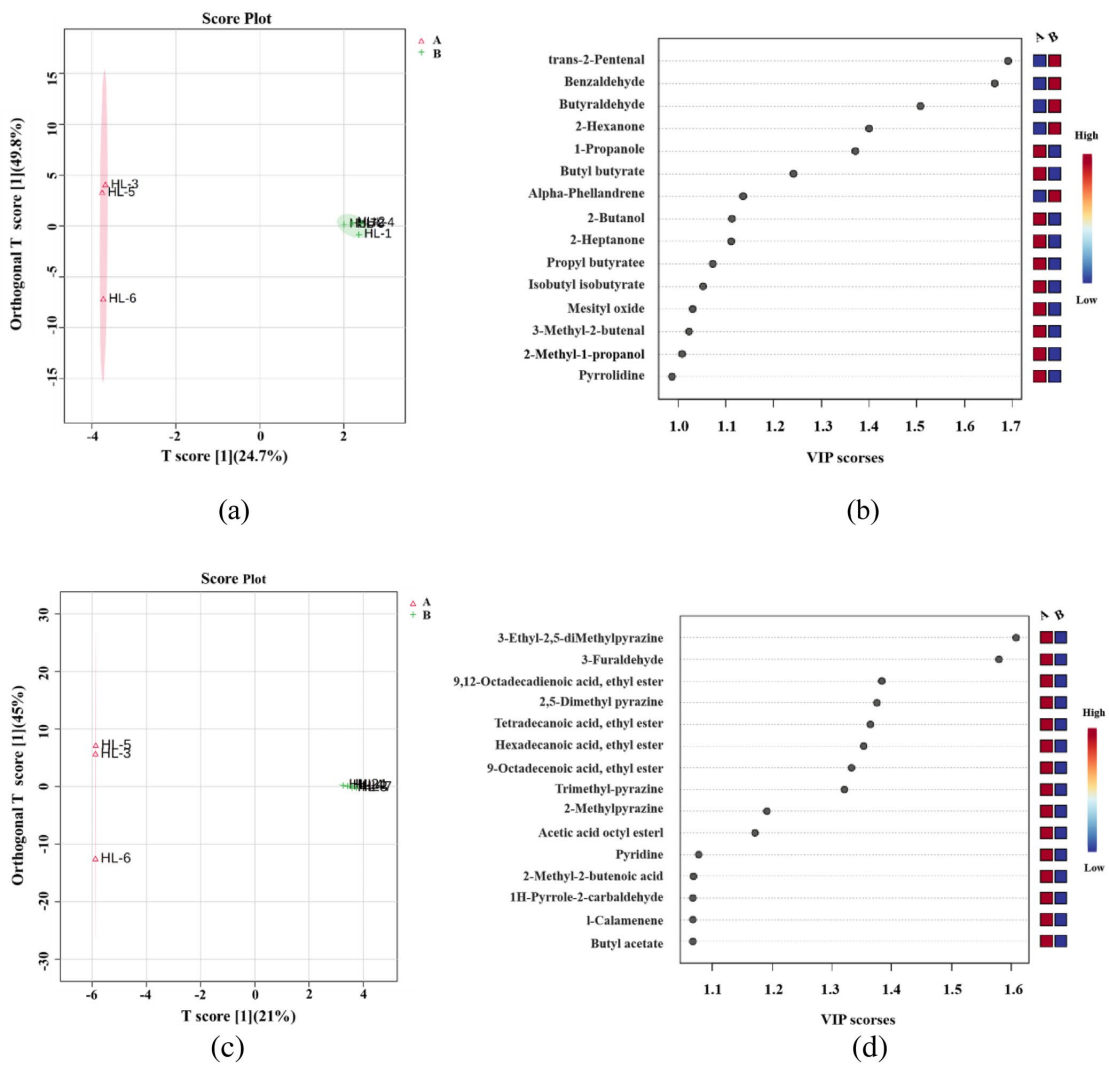
OPLS‐DA and VIP of characteristic volatile components in different Fenugreek Tincture samples. (a) chart of OPLS‐DA for GC‐IMS; (b) chart of VIP for GC‐IMS; (c) chart of OPLS‐DA for SPME‐GC–MS; (d) chart of VIP for SPME‐GC–MS. (Considering the display effect, the picture shows only the first 15 compounds).

Samples with darker appearance color (HL‐3, HL‐5, and HL‐6) and samples with lighter appearance color (HL‐1, HL‐2, HL‐4, HL‐7, and HL‐8) were classified into groups A and B, respectively, which were then subjected to supervised OPLS‐DA. By removing variables unrelated to sample categorization, OPLS‐DA can provide visualized complicated data prediction and analysis, which can precisely identify the differences between the two groups comparing to PCA. As shown in Figure 9a,c, all the samples fell inside the 95% confidence interval. Sample distribution of Group A presents in the left quadrant while that of Group B presents in the right quadrant. This indicates that the two sample groups can be distinguished effectively. The principal component scores, which display the variations between groups, are indicated by the horizontal coordinates. The orthogonal component scores, which display the variations between samples within each group, are indicated by vertical coordinates. Within group A, there are significant variations between samples while less discovered within group B.

Variable Importance Projection (VIP) approach can be used to screen key substance fractions that are important for fenugreek tincture samples from different manufacturers or production process. Key variable analysis is evaluated by VIP values obtained from OPLS‐DA. If the variable has a VIP‐value over 1, this it can be considered to have a noteworthy contribution to the model. As displayed in Figure 9d,b, red color represents high concentration while blue color represents low concentration. For GC‐IMS VIP scores shown in Figure 9b, a total of 12 components with VIP values larger than 1 were screened. When combining with ANOVA test, benzaldehyde, trans‐2‐pentenal, 2‐hexanone, and n‐butyraldehyde were determined to have significant differences (*p* < 0.05), while butyl butyrate, propyl butyrate, and n‐propanol only have some differences (*p* < 0.1). A total number of 81 components with VIP values greater than 1 were screened from SPME‐GC–MS analysis as shown in Figure 9d. According to ANOVA test, 3‐ethyl‐2,5‐dimethylpyrazine and 3‐furaldehyde were found to be significantly different (*p* < 0.05) while 2,5‐dimethylpyrazine, ethyl myristate, and ethyl linoleate had certain difference (*p* < 0.1). Therefore, it can be postulated that the 12 components listed above have significant influence on the categorization of the tested fenugreek tincture samples. These components are the primary differential chemical markers that are responsible for the variations in the volatile composition and the appearance colors. It can provide some indications for distinguishing fenugreek tincture samples from different manufacturers and production processes (Huang 2005).

#### 3.4.3 Analysis of Odor Activity Value (OAV) of Differential Volatile Components

In addition to their content, each volatile component's contribution to the overall aroma of fenugreek tincture is closely correlated with their odor thresholds. In order to further identify the active flavor ingredients in fenugreek tincture samples, OAVs of the 12 screened differential chemical markers were calculated to evaluate their contribution to the overall flavor. Table 3 listed nine components that have an OAV greater than 1, which are benzaldehyde, trans‐2‐pentenal, 2‐hexanone, n‐butyraldehyde, butyl butyrate, propyl butyrate, 3‐ethyl‐2,5‐dimethylpyrazine, 2,5‐dimethylpyrazine, and ethyl linoleate. These components can be determined as the characteristic flavor components that can affect overall flavor. The most prevalent species are aldehydes and esters, which further suggests that they are the primary contributors for the distinctive flavor of fenugreek tincture. These two key flavor components were found to have the highest and lowest concentration in sample HL‐6 and HL‐1, respectively, which verifies the different flavor from the tested samples. Within the compounds listed in Table 3, some of the components have OAVs even over 10,000. Benzaldehyde is the main characteristic aroma component of roasted nuts, with a pleasant almondy and slightly fruity aroma. The scent of trans‐2‐pentenal is unique and potent; the smell of n‐butyraldehyde is grassy; esters smell like fruit, while pyrazines smell greenish, nutty, and legume‐like. These constituents make a significant contribution for shaping the characteristic flavor of fenugreek tincture. Therefore, these compounds are considered as the key flavor components for fenugreek tincture. Even if the chemical compositions of the samples differ, the OAVs of key flavor components can still dominate the primary aroma characteristics. For example, in Figure 6, the composition ratios of HL‐1, 2, 3, and 5 vary greatly, but the OAVs of their key flavor components, such as benzaldehyde and n‐butyraldehyde, still show similar distributions. This results are similar for aroma profiling of HL‐1, 2, 3, and 5. This outcome is in line with the DSA results. OAV evaluations provides a theoretical support for flavor identification of fenugreek tincture samples by elucidating the impact of each component on the overall flavor. It demonstrates that the characteristic flavor evaluation of fenugreek tincture samples can be validated by GC‐IMS/SPME‐GC–MS techniques coupled with OAV calculations, which provide a new strategy for the study of flavor characterization of natural extracts.

**TABLE 3:** Threshold and OAV of volatile flavor compounds in different fenugreek tincture samples (Gemert 2015, 2018).

Compound	Threshold (mg/kg)	HL‐1	HL‐2	HL‐3	HL‐4	HL‐5	HL‐6	HL‐7	HL‐8
Benzaldehyde	0.7508	127.87	136.51	137.75	138.92	139.25	103.58	141.06	133.91
(E)‐2‐pentenal	0.98	0.68	0.62	0.75	0.68	0.65	10.03	0.66	0.68
2‐Hexanone	0.56	15.67	23.02	13.33	15.23	13.73	42.54	23.25	22.85
Butanal	0.002	15,925.19	19,814.15	16,217.82	11,592.41	17,425.22	15,719.68	19,599.44	19,977.24
Butanoic acid butyl ester	0.4	2.27	1.06	8.28	1.84	1.33	29.23	1.62	1.40
Propyl butyrate	0.018	339.19	32.39	27.07	26.79	2297.33	824.79	39.97	39.09
1‐Propanol	8.5056	0.10	0.08	0.30	0.08	0.08	0.58	0.05	0.06
3‐Ethyl‐2,5‐dimethyl‐pyrazine	0.0086	0	0	2041.860	0	1374.42	77.91	0	124.42
3‐Furaldehyde	9.562	0	0	0	0	0.77	0.54	0	0
2,5‐Dimethyl‐pyrazine	1.75	0	8.34	42.27	1.27	22.03	0	4.27	3.85
Tetradecanoic acid ethyl ester	4	0	0	0	0	0.13	0.41	0	0
Linoleic acid ethyl ester	4	0	0	0	0.32	1.03	2.85	0	0

#### 3.4.4 Correlation Analysis Between OAV Test and Sensory Characteristic Flavor

**FIGURE 10:**
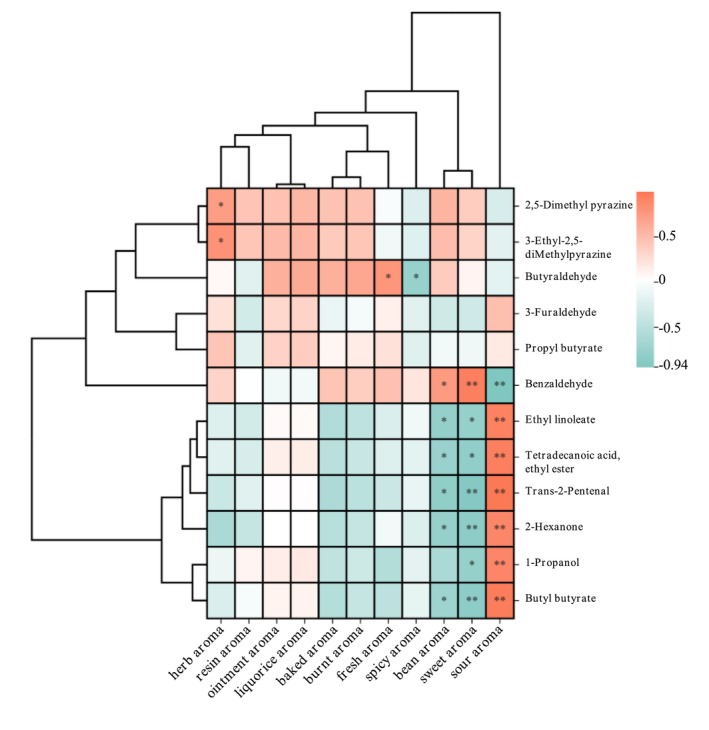
Spearman's correlation heatmap between the OAV and the aroma attribute (*p < 0.05, **p < 0.01).

Correlation clustering heatmaps were also generated to investigate the link between the sensory characteristic flavor and OAVs of the differential components. Each column in the heat map (Figure 10) represents a characteristic flavor indicator, while each row indicates an OAV. The correlation between the OAV and flavor is represented by the color of each block in the heat map. The color was determined by the correlation coefficient. Red color indicates positive correlation, and green color indicates negative correlation, while darker color indicates stronger correlation (* indicates *p* < 0.05, ** indicates *p* < 0.01). Combining with Table 4, the index of burnt, baked, and herbal aroma are positively correlated with benzoaldehyde, n‐butyraldehyde, propyl butyrate, 3‐ethyl‐2,5‐dimethylpyrazine, and 2,5‐dimethylpyrazine, which suggests that this group of five components have a predominant contribution to these aromas. N‐butanal is observed to have an OVA over 10,000, which shows a significant positive correlation with fresh aroma and a negative correlation with spicy aroma. Benzaldehyde was observed to have high concentration and strong aroma vitality, which is also significantly positive correlated to sweet aroma. Therefore, benzaldehyde plays a significant role in contributing to the sweet aroma of fenugreek tincture. Herbal aroma is found to have a positive correlation with 2,5‐dimethylpyrazine and 3‐ethyl‐2,5‐dimethylpyrazine. This suggests that both components are the major contributors to the herbal aroma of the samples. Sour aroma is significantly positively correlated to ethyl linoleate, ethyl myristate, trans‐2‐pentenal, 2‐hexanone, n‐propanol, and butyl butyrate. The findings suggest a strong correlation between sensory characteristic flavor and volatile components composition of the samples from various manufacturers. These findings are in line with the nine key flavor components identified previously as shown in Table 3. This suggests that n‐butanal, 3‐ethyl‐2,5‐dimethylpyrazine, and 2,5‐dimethylpyrazine are the major contributors for the primary flavor of fenugreek tincture, which is a complex aroma with mainly burnt and herbal aroma.

**TABLE 4:** Correlation of differential compounds with aroma.

Compound	Aroma association						
	Burnt aroma	Baked aroma	Herbal aroma	Fresh aroma	Spicy aroma	Sweet aroma	Sour aroma
Benzaldehyde	+	+	+			+	
(E)‐2‐pentenal							+
2‐Hexanone							+
Butanal	+	+	+	+	−		
Butanoic acid butyl ester	+	+	+				+
Propyl butyrate	+	+	+				+
1‐Propanol							
3‐Ethyl‐2,5‐dimethyl‐pyrazine	+.	+	+				
3‐Furaldehyde							
2,5‐Dimethyl‐pyrazine	+	+	+				
Tetradecanoic acid ethyl ester							+
Linoleic acid ethyl ester							+

## 4 Conclusion

This study examines the variations in the key volatile flavor components of several fenugreek tincture samples from various manufacturers and their relationship with flavor characterization using GC‐IMS, SPME‐GC–MS, OAV, and DSA. The results of the sensory evaluation showed that the samples were overall dominated by burnt and herbal aroma, supplemented by sweet, hay, and balsamic aroma. There were 148 volatile components identified from both techniques with 37 found by GC‐IMS and 114 by SPME‐GC–MS. Aldehydes, esters, and lactones were found to be the most diverse groups across all samples. The differences in the volatile components compositions of fenugreek tincture samples presented quite different flavors. There are nine key flavor components that were determined by OAV evaluation. When it comes to burnt, baked, and herbal aroma indicators, benzaldehyde, n‐butyraldehyde, propyl butyrate, 3‐ethyl‐2,5‐dimethylpyrazine, and 2,5‐dimethylpyrazine showed positive correlations. Significant positive correlation was observed between n‐butyraldehyde and fresh aroma. Benzaldehyde was found to have significant positive correlation with sweet aroma. Both 2,5‐dimethylpyrazine and 3‐ethyl‐2,5‐dimethylpyrazine exhibited a significant correlation with herbal aroma. The primary distinctive aroma of fenugreek tincture is composed by all these highly associated volatile components. This study elaborates on the intrinsic association between key flavor components and sensory characteristic flavor, as well as the contributions of these components to the overall flavor of fenugreek tincture. This work can provide a technical views for future research on the flavor and sensory attributes of fenugreek tincture.

## Author Contributions

**Hua Zhang:** data curation (equal), investigation (equal), methodology (equal), software (equal), writing – original draft (equal). **Haifeng Shen:** data curation (equal), investigation (equal), methodology (equal), software (equal), writing – original draft (equal). **Yuanqing Ye:** investigation (equal), writing – review and editing (equal). **Fan Cao:** investigation (equal), writing – review and editing (equal). **Jiale Ren:** validation (equal). **Huaiyuan Zhu:** validation (equal). **Bo Chi:** validation (equal). **Huiyun Liao:** conceptualization (equal), funding acquisition (equal), writing – review and editing (equal). **Feng Li:** conceptualization (equal), funding acquisition (equal), writing – review and editing (equal).

## Ethics Statement

The authors have nothing to report.

## Consent

The authors have nothing to report.

## Conflicts of Interest

The authors declare no conflicts of interest.

## Data Availability

Data will be made available from the corresponding author upon reasonable request.
